# Marine Sponge/CuO Nanocrystal: A Natural and Efficient Catalyst for Sulfonamides Synthesis

**Published:** 2012-10-07

**Authors:** Mohamad Reza Shushizadeh, Azar Mostoufi, Marjan Fakhrian

**Affiliations:** 1 Research Center of Marine Pharmaceutical Science, Ahvaz Jundishpur University of Medical Science, Ahvaz, IR Iran; 2 Islamic Azad University, Science and Research Branch-Khoozestan, Ahvaz, IR Iran

**Keywords:** Marine, Sponge, Synthesis, Nanoparticles, Sulfonamides

## Abstract

**Background:**

Marine sponge/nano-CuO as a natural catalyst efficiently catalyzed the Sulfonylation reaction of p-chlorobenzene sulfonyl chlorides with amines in order to prepare sulfonamides. The advantages included use of a natural catalyst, ease of handling, requirement of a very small amount of catalyst, mild reaction condition and appropriateness to high yield.

**Objectives:**

The current study aimed to look for a solid support reaction and to develop a general, mild and novel method in order to synthesize sulfonamides in the absence of a strong base, it was found that marine sponge/nano-CuO is a natural and efficient catalyst for this method at room temperature.

**Materials and Methods:**

The reaction was carried out simply by addition of amine and p -chlorobenzene sulfonyl chloride to the mixture of Marine sponge powder/nano-CuO in acetonitrile at room temperature. Then the reaction mixture was extracted by CH2Cl2 and was dried over anhydrous magnesium sulfate. Evaporation of the solvent afforded the products.

**Results:**

In this method several derivatives of sulfonamide underwent the reaction of different amines with p-chlorobenzene sulfonyl chloride in the presence of marine sponge/nano-CuO in CH3CN are synthesized.

**Conclusions:**

In conclusion, a new, natural and efficient marine catalyst, and a marine sponge/nano-CuO were developed to synthesize sulfonamide derivatives in CH3CN in 75–93% yields. This method was applied to a wide range of aromatic and aliphatic amines under mild conditions.

## 1. Background

The development of simple, natural, efficient and environmentally-benign chemical processes or methodologies for widely used organic compounds is greatly demanded. Sulfonamides are extremely useful pharmaceutical compounds because they exhibit a wide range of biological activities such as anticancer, anti-inflammatory and antiviral functions (1). Furthermore, sulfonamides have been used as protecting groups of OH or NH functionalities for easy removal under mild conditions (2). Even though many synthetic methods have been reported (3), the sulfonylation of amines with sulfonyl chlorides in the presence of a base is still being used as the method of choice because of its high efficiency and simplicity of the reaction (4). However, this approach is limited by the formation of undesired disulfonamides with primary amines and by harsh reaction conditions needed for less nucleophilic amines such as anilines (5). Additionally, side reactions take place in the presence of a base. Metal and metal oxides have been used for the catalytic sulfonylation of amines and alcohols; however, it requires a longer reaction time and stringent reaction conditions (6-8).

Marine sponges are known as a prolific source of biologically active and structurally unique metabolites. They are known to produce a large number and a diversity of secondary metabolites. Until now, more than 5000 different compounds have been isolated from about 500 species of sponges (9). The chemical nature of metabolites isolated from marine sponges has been extensively reviewed by several authors (10). As there was no report of marine sponges of Iranian coast of Persian Gulf, shallow sponges (Desmospongea sp.) of Qeshm and Bushehr Islands in offshore zone which can be the source of new biological active compounds were studied. Marine sponges occupy a preeminent position among the various groups of organisms. They are a unique group of sedentary organisms from which several novel natural products are reported, many of which have useful biological activities. In organic chemistry, these sponges are important and are an optical active source for catalytic reactions such as oxidation, reduction and etc. (11). The chiral non-racemic catalyst, marine sponge, which has optical active compounds such as alkaloids, and terpenoids, is a good catalyst for solid support reactions and induction of chirality into desired products with excellent enantioselectivities. Marine sponge can activate the C-N bond for nucleophilic addition such as sulfonylation reaction of amines with high and predictable asymmetric induction, and is easily removed from the product. Therefore, the search continues for a better catalyst in sulfonylation reaction of amines in terms of operational simplicity, with greater yields.

## 2. Objectives

The current study aimed to look for a solid support reaction (12) and to develop a general, mild and novel method in order to synthesize sulfonamides in the absence of a strong base. The current study tried to report a simple and efficient method for the sulfonamides synthesis, in the presence of marine sponge/nano-CuO as a natural catalyst as indicated in Figure 3.

## 3. Materials and Methods

### 3.1. Reagents and Materials

All starting materials were purchased from Merck and Aldrich Companies. The IR spectra were recorded on a Perkin-Elmer RXI infrared spectrometer. 1H NMR spectra were recorded with a 400 MHz Broucker FT-NMR spectrometer. TLC accomplished the purity of substrates and reactions monitored on silica gel polygram SIGL/UV254 plates.

### 3.2. Preparation of Marine Sponge Powder

The Samples, marine sponge (Demospongiae sp.), were collected in May 2010 at a depth between 5 and 10 m in the Nakhiloo Island, Bushehr, Iran (North coast of Persian Gulf) and were washed several times using deionized water to remove extraneous and salts. They were then dried in an oven at 60 °C for 48 h. The dried marine sponge chopped, sieved and the particles with an average size of 0.5mm were used for base catalyzed experiments. Identification of sponges was kindly carried out by Dr. Sayed Mohammad Bagher Nabavi from Khoramshahr Marine Science and Technology University. Figure 1a shows the species of marine sponge investigated in this study as Demospongiae sp., which has siliceous (SiO_2_) spicules, Figure 1b.

The species of marine sponge and its spicules

**Figure 1a:**
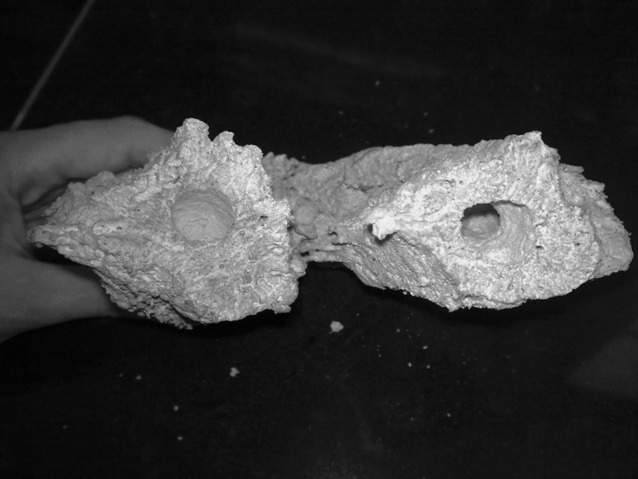
Marine sponge (Demospongiae sp.)

**Figure 1b:**
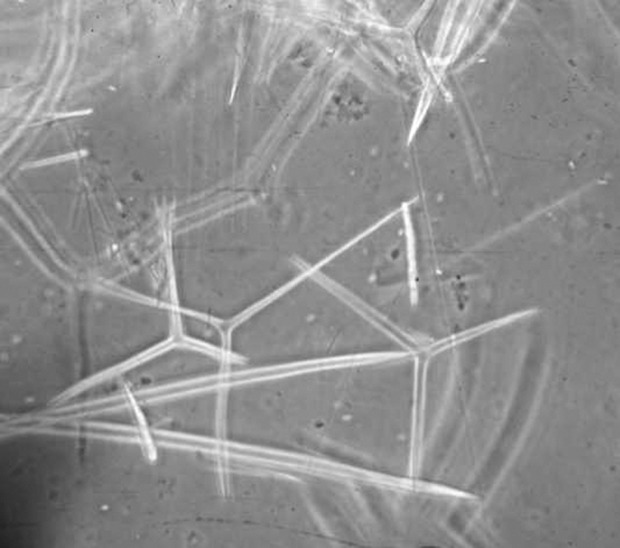
Siliceous spicules

### 3.3. Preparation of Nano-CuO

**Figure 2:**
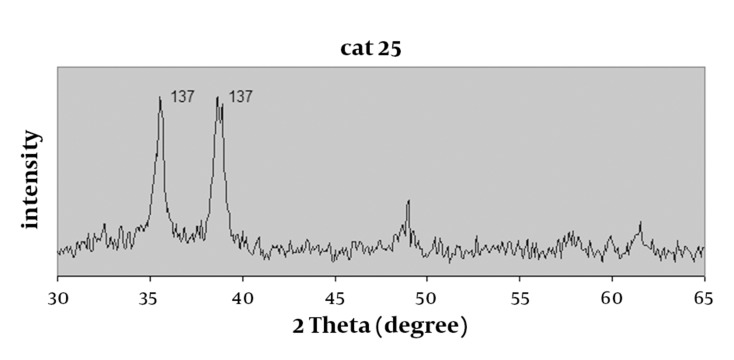
XRD spectrum of Nano-CuO

300mL of 0.02M Cu (NO_3_)_2_ solution was prepared by dissolving Cu(NO_3_)_2_·3H_2_O in deionized water. The solution was added into a round-bottom flask equipped with a refluxing device. The Cu (NO_3_)_2_ solution was kept at 60 ◦C by vigorous stirring; then 0.50 g of solid NaOH (platelets) was rapidly added into the solution, where a large amount of blue or black precipitate was simultaneously produced and maintained at the crystallization temperature for 10 min. Next, the precipitate was heated at 100 °C for another 10 min. During this process, the initial blue color of the precipitate was gradually turned into black. When all reactions were completed, the resulting product was centrifuged, washed with water and ethanol several times, and dried in air at room temperature (13). Crystal structure of nano-CuO was identified by XRD spectrum displayed in Figure 2.

### 3.4. General Procedure

2 mmol Amine and 2 mmol p -chlorobenzene sulfonyl chloride were added to the mixture of 0.09 g Marine sponge powder , 0.01 g nano-CuO and 10 mL of acetonitrile ,and then it was stirred magnetically at room temperature. The progress of the reaction was monitored by thin-layer chromatography (TLC). Then the reaction mixture was extracted with 2×25 mL CH_2_Cl_2_. The combined solutions were dried over anhydrous magnesium sulfate. Evaporation of the solvent afforded the products which are shown in Table 1. The structures of the products were characterized by their melting points and 1H NMR and IR spectral data.

**Table 1:** Sulfonylation of p-anisidine with p-toluene sulfonyl chloride in the presence of 0.1 g marine sponge/nano-CuO with different solvents

	Solvent	Time, h	Yield, %
1	THF	5	60
2	CH_2_Cl_2_	2.5	80
3	CHCl_3_	1.5	75
4	EtOAc	3	70
5	CH_3_CN	50(min)	93

## 4. Results

**Figure 3:**
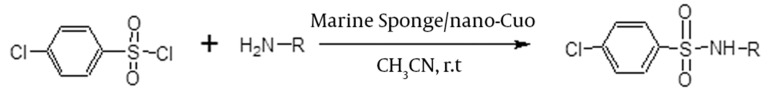
Sulfonylation of amines in the presence of marine sponge/nano-CuO

Sulfonamide derivatives simply synthesized by sulfonylation of amines in the presence of marine sponge/nano-CuO as natural catalyst. Figure 3 displays the reaction of sulfonyl chlorides with amines for production of sulfonamides. In order to find out the most effective sulfonylation, p-anisidine was chosen as a model substrate. It was treated by 2 mmol of p-chlorobenzene sulfonyl chloride in the presence of 0.1 g of marine sponge/nano-CuO powder in various solvents at room temperature as shown in Table 1. The reactions in THF, CH2Cl2, CHCl3, and EtOAc were found less effective, Table 2, entries 1–4. Then the sulfonylation of p-anisidine by p-chloro sulfonyl chloride in the presence of the CH3CN solvent to get 93% yield was carried out, Table 2 entry 5.

**Table 2:** Sulfonylation of p-anisidine with p-chloro sulfonyl chloride in the presence of different catalyst with CH3CN as a solvent

	Catalysts	Mmol, (mg)	Time, h	Yield, %
1	Marine sponge /nano-CuO	0.2 (100)	50(min)	93
2	CuO	0.2 (16)	1	92
3	MgO	0.2 (8)	3	50
4	CaO	0.2 (11.2)	4	51

In order to find out the most effective catalyst for sulfonylation, various catalysts were employed during the sulfonation of 1:1 equimolar p-anisidine with p-chlorobenzene sulfonyl chloride at room temperature, Table 2. According to the results, marine sponge/nano-CuO was found to be the most efficient catalyst. Several derivatives of sulfonamide which underwent the reaction of different amines with p-chlorobenzene sulfonyl chloride in the presence of marine sponge/nano-CuO in CH3CN were synthesized, Table 3.

**Table 3:**
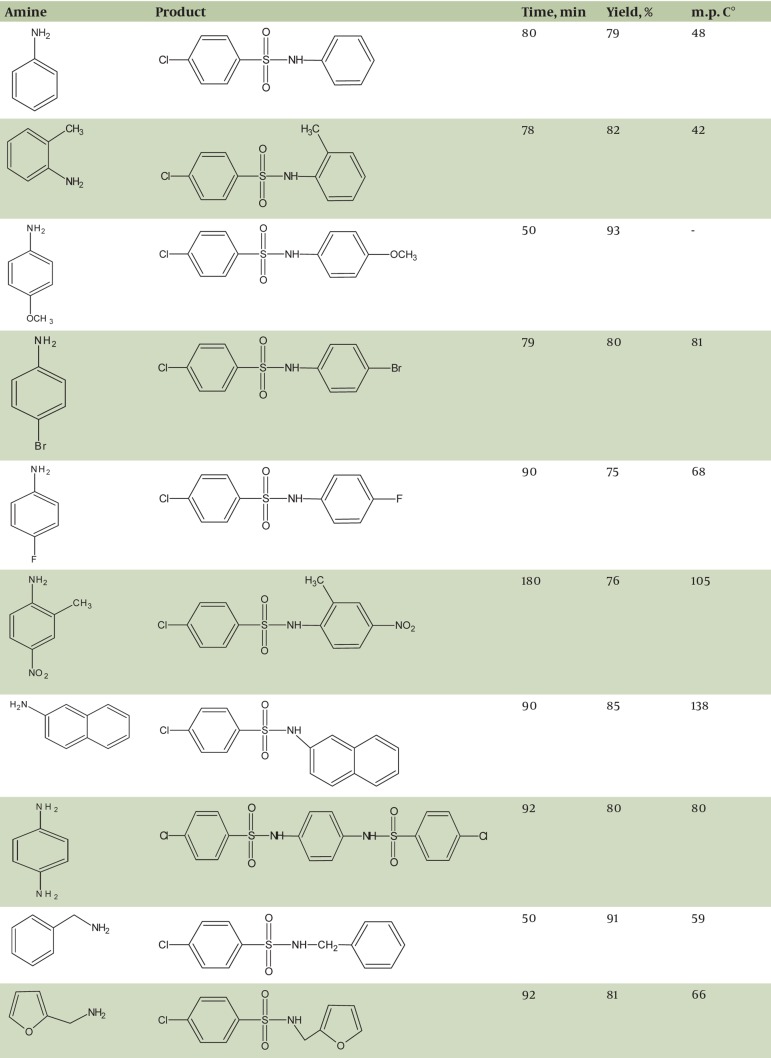
Sulfonamides synthesis by the reaction of different amines with p-chloro sulfonyl chloride in the presence of marine sponge/nano-CuO in CH3CN

Aromatic amines were sulfonylated under the CH_3_CN solvent at room temperature with excellent yields, Table 3, entries 1–8. Aromatic amines with an electron-donating group in Table 3, entries 2–3 showed similar reactivity, whereas those with an electron-withdrawing group in Table 3, entries 4–6 showed somewhat lower reactivity. Aliphatic amines which were also sulfonylated under similar conditions gave excellent yields , Table 3, entries 9–10.

### 4.1. Characterization of products

Selected spectral data for products shown in Table 3 are given below:

4-Chloro-N-phenyl benzene sulfonamide-(entry 1): IR(cm^-1^, KBr): 3250 (-NH), 1541 (C=C), 1336, 1161( SO_2_ ), 826752 , (aromatic); ^1^HNMR (400 MHz, CDCl_3_, TMS, δ ppm): 7.07-7.28 (3H, m), 7.3-7.41 ( 2H, m), 7.49-7.57 (2H, d), 7.64-7.8 (2H, d).

4-Chloro-N-(4-methoxyphenyl)benzenesulfonamide-(entry 3):IR(cm^-1^,KBr) :3231(-NH), 1550 (C=C), 1334, 1160( SO_2_ ), 819, 751 (aromatic); ^1^HNMR (400 MHz, CDCl_3_, TMS, δ ppm): 3.73 (3H, s), 6.74-6.81 (2H, d), 6.99-7.1 (2H, d), 7.19-7.4 (2H, d), 7.61-7.79 (2H, d).

4-Chloro-N-(4-fluorophenyl)benzenesulfonamide-(entry 5):IR(cm^-1^,KBr) :3247(-NH), 1541 (C=C), 1334, 1160( SO_2_ ), 830757 , (aromatic); ^1^HNMR (400 MHz, CDCl_3_, TMS, δ ppm): 6.82-6.90 (2H, d), 6.94-7.1 (2H, d), 7.2 (1H, bs), 7.3-7.43 (2H, d), 7.51-7.67 (2H, d).

4-Chloro-N-(2-methyl-4-nitrophenyl)benzenesulfonamide-(entry 6):IR(cm^-1^,KBr): 3240 (-NH), 1543 (C=C), 1334, 1160( SO_2_ ), 830757 , (aromatic); ^1^HNMR (400 MHz, CDCl_3_, TMS, δ ppm): 2.2 (3H, s), 7.2-7.6 (3H, m), 7.94-8.01 (4H, m).

4-Chloro-N-(2-naphthyl)benzenesulfonamide-(entry 7):IR(cm^-1^,KBr) :3230(-NH), 1585 (C=C), 1334, 1160( SO^2^ ), 860, 730 (aromatic); ^1^HNMR (400 MHz, CDCl_3_, TMS, δ ppm): 7.08 (1H, bs), 7.28-7.49 (4H,m), 7.45-7.49 (3H, m), 7.6-7.8 (4H, m)

4-Chloro-N-(benzyl)benzenesulfonamide-(entry 9):IR(cm^-1^,KBr) :3201(-NH), 1560 (C=C), 1334, 1160( SO_2_ ), 821742 , (aromatic); ^1^HNMR (400 MHz, CDCl_3_, TMS, δ ppm): 4.11 (2H, d), 7.16-7.32 (5H, m), 7.41-7.65 (2H, d), 7.75 (2H, d).

## 5. Discussions

**Figure 4:**
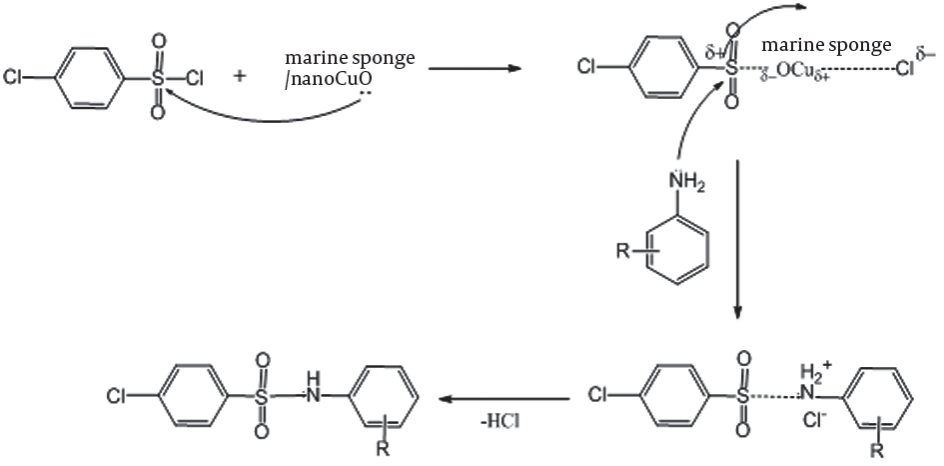
Mechanism of sulfonamides synthesis in the presence of marine sponge/nano-CuO

In conclusion, a new natural and efficient marine catalyst, and a marine sponge/nano-CuO were developed to synthesize sulfonamide derivatives in CH3CN in 75–93% yields. This method was applied for a wide range of aromatic and aliphatic amines under mild conditions. Additionally, the key note of this synthetic protocol was the absence of a strong base in the reaction. The plausible mechanism of the reaction is shown in Figure 4.
